# Mechanisms of feature binding in visual working memory are stable over long delays

**DOI:** 10.1167/jov.21.12.7

**Published:** 2021-11-16

**Authors:** Georgina Brown, Iham Kasem, Paul M. Bays, Sebastian Schneegans

**Affiliations:** 1 Department of Psychology, University of Cambridge, Downing Street, Cambridge, UK

**Keywords:** visual working memory, feature binding, swap errors, neural population model

## Abstract

The ability to accurately retain the binding between the features of different objects is a critical element of visual working memory. The underlying mechanism can be elucidated by analyzing correlations of response errors in dual-report experiments, in which participants have to report two features of a single item from a previously viewed stimulus array. Results from separate previous studies using different cueing conditions have indicated that location takes a privileged role in mediating binding between other features, in that largely independent response errors have been observed when location was used as a cue, but errors were highly correlated when location was one of the reported features. Earlier results from change detection tasks likewise support such a special role of location, but they also suggest that this role is substantially reduced for longer retention intervals in favor of object-based representation. In the present study, we replicated the findings of previous dual-report tasks with different cueing conditions, using matched stimuli and procedures. Moreover, we show that the observed patterns of error correlations remain qualitatively unchanged with longer retention intervals. Fits with neural population models demonstrate that the behavioral results at long, as well as short, delays are best explained by memory representations in independent feature maps, in which an item's features are bound to each other only via their shared location.

## Introduction

Feature binding in visual working memory is the mechanism that allows us to memorize not only separate visual features, such as colors, shapes, and orientations, but also their specific conjunctions that characterize objects in the visual world (Treisman, 1996). The properties and limitations of this mechanism have been a topic of active research for more than two decades (see Schneegans & Bays, 2019, for review). The seminal study of Luck and Vogel (1997) popularized slot-based models, in which bound object representations that encompass all features of a stimulus are the natural units of working memory. This conceptualization was based on results from change detection experiments, indicating that working memory capacity was limited only in the number of objects to be memorized, rather than the number of constituent visual features.

Subsequent research showed that such object-based capacity limits only captured behavioral performance when the objects were characterized by a combination of different (and simple) features, but not when they combined two features of the same kind, such as two different colors (Wheeler & Treisman, 2002; Xu, 2002). These findings instead pointed toward separate feature stores with independent capacity limits. The object file theory (Kahneman et al., 1992; Wheeler & Treisman, 2002; Treisman & Zhang, 2006) proposes that such separate stores for unbound features coexist with a limited set of object files, bound object representations that are formed by sequentially focussing attention on individual stimuli.

Whereas classical change detection tasks have to rely on comparisons of performance between different experimental conditions to assess memory for features and bound objects, analogue report paradigms can elucidate binding mechanisms more directly. In this type of task, participants view a memory sample array and then report a feature of a cued item on a continuous scale (Wilken & Ma, 2004; Zhang & Luck, 2008). Two studies (Bays et al., 2011b; Fougnie & Alvarez, 2011) independently of each other extended this paradigm into a dual-report task, in which participants reported two features of the cued item. In the experiments, participants viewed an array of five or six colored, oriented bars or triangles. After a brief retention interval, they were cued with the location of one of the sample items to report both its color and its orientation. Using mixture models and classification of response errors in single trials, both studies concluded that failures to recall the two features occurred nearly independently of each other.

This finding is in clear conflict with slot models, which predict that whole objects with all their features should be either remembered or not. However, the results are also inconsistent with predictions of the object file theory. To accurately recall either of the two features (color and orientation) in this task, the feature has to be bound to the stimulus location that is used to indicate the target item. So, rather than a combination of a few complete object representations and additional unbound features as proposed by the object file theory, working memory seems to contain a collection of partially bound representations, with colors and orientations of different objects encoded with their associated location.

A range of findings from behavioral and imaging studies indicate that location has a special role in working memory and feature binding. Spatial memory is very precise and object location is a particularly effective cue for retrieving other visual features (Rajsic et al., 2017; Schneegans & Bays, 2017). Object location can be decoded from neural activity (Foster et al., 2017) and attention is drawn to it when a memorized item is cued (Kuo et al., 2009), even when location is entirely task-irrelevant. Moreover, Treisman and Zhang (2006) have shown that task-irrelevant location changes affect performance and response biases when participants have to detect changes in color-shape binding (see also Logie et al., 2011), leading these authors to propose that binding between nonspatial features is at least in part mediated by location.

The role of location in feature binding was addressed in another dual-report experiment (Schneegans & Bays, 2017, replicated and extended by Kovacs & Harris, 2019), in which participants again had to remember an array of colored oriented bars. They were then cued either with the color or the orientation of one sample item, and had to report its location together with the other nonspatial feature. Under these conditions, strongly correlated response errors were observed. More specifically, when participants erroneously reported the location of a nontarget item, they tended to also report the other feature of that spatially selected item, whereas the response was indistinguishable from guessing when compared with the feature of the true target item. This phenomenon was observed even when the location was reported after the other feature, demonstrating that it was not the location report itself that drove this behavior.

Schneegans and Bays (2017) used neural population models with conjunctive coding of multiple features (Matthey et al., 2015) to explain the observed patterns of error correlations, and found that they were well-captured by a model with separate feature maps over visual space. In this model, the different features can be retrieved from their respective map independently of each other when cued with the target item's location. However, when cued with, for example, the orientation of one item, the only way to retrieve the associated color is to first determine the location where the cue feature appears in the orientation map and then use that to retrieve the color. When an error occurs in the first step (e.g., a nontarget item with a similar orientation as the target is selected because of noise in encoding and decoding of features), then this incorrect location will also be used to retrieve the color value, yielding correlated response errors of the form observed in the behavioral data.

Feature maps over visual space are well-established in models of visual attention and visual search (Itti & Koch, 2001; Hamker, 2004), but they are not widely considered to be the substrate of visual working memory. For instance, in Treisman's feature integration theory, it is assumed that the attentional selection of an individual object is realized through activation of a spatial region across different feature maps (Treisman & Gelade, 1980; Treisman, 1988), but the object's features are then consolidated in the form of object files (Kahneman et al., 1992; Wheeler & Treisman, 2002).

Indeed, there is some evidence that the special role of location may only extend from perception to the earlier stages of working memory. In the study of Treisman and Zhang (2006), the effect of task-irrelevant location changes was strongest when the retention interval between sample and test array was short (0.1 s or 0.9 s), and was substantially diminished at longer intervals of 3 s and 6 s. Similarly, a study by Logie et al. (2011) found a substantial disruptive effect of task-irrelevant location changes when retention intervals up to 1.5 s were used, but the effect was no longer significant for longer retention intervals. Moreover, this study found that performance in the condition with random location changes improved with longer retention intervals, indicating that the mitigation of the disruptive effect is strong enough to cancel out the expected decrease in performance with longer delays.

The previous dual-report experiments used relatively brief retention intervals of approximately 1 s. It is therefore plausible that the binding mechanism that was proposed based on these results only applies to the early stages of visual working memory, and does not capture behavior when items have to be memorized over longer durations. Moreover, the findings of largely independent response errors when cued with location versus correlated errors when cued with a nonspatial feature were obtained in separate studies, using similar, but not identical stimuli and procedures. That details of the experiment design can make a substantial difference is demonstrated by the study of Gajewski and Brockmole (2006), in which participants viewed an array of colored shapes and had to verbally report the (categorical) color and shape of an item cued by its location. Unlike the later dual-report experiments, the study found high correlations between response errors for the two features. It was later shown that this finding could be attributed to the brief presentation time and large eccentricity of stimuli in this study, which likely incentivized participants to focus their attention on a subset of stimuli (Peich et al., 2013).

The objective of the present study is two-fold: First, we aim to replicate the findings of earlier dual-report experiments for short retention intervals with both location cues (Bays et al., 2011b; Fougnie & Alvarez, 2011) and nonspatial feature cues (Schneegans & Bays, 2017), using matched stimulus displays and response procedures. We will also apply the different forms of analysis used by the previous studies to both experimental conditions, namely, mixture model fits to estimate error correlations and model comparison between neural population models implementing different forms of feature binding.

The second aim is to investigate whether the pattern of error correlations changes with longer retention intervals, which would indicate a change in the underlying format of working memory representations. To test this hypothesis, we interleave two different retention intervals in each dual report task. The shorter one matches the duration used in earlier dual report tasks, whereas the latter was chosen to be in a range for which change detection experiments observed a substantially decreased effect of task-irrelevant location changes. To preview our results, we found matching patterns of error correlations in both delays, which were consistent with those reported in earlier studies.

## Experiment 1

Experiment 1 used a dual-report task in which participants had to report the color and orientation of a sample stimulus cued by its location. We aimed to replicate the findings of Bays et al. (2011b) and Fougnie and Alvarez (2011) regarding response error correlations, and extend them to longer retention intervals by using two different delay durations. We used a stimulus display more similar to that of Schneegans and Bays (2017), but with stimulus sizes intermediate between that study and Bays et al. (2011b).

### Methods

#### Participants

Twelve participants (five female, seven male, aged 18–30 years) completed the experiment after giving informed consent in accordance with the declaration of Helsinki. All participants showed normal color vision and reported normal or corrected-to-normal visual acuity. Participants were recruited via an online sign-up system or by word of mouth, and were compensated with £10 per hour for their time. All participants were naive to the experimental hypothesis. The sample size was chosen based on the previous studies, which had found clear and consistent evidence for independent response errors with sample sizes of 10 and 12, respectively.

#### Apparatus

Stimuli were presented on a 27-inch LCD monitor with a refresh rate of 120 Hz (Asus Swift PG279). Participants were seated in front of the monitor at a viewing distance of 60 cm, with their head position stabilized by a forehead and chin rest. Participants’ gaze direction was monitored using an infrared video-based eye tracker (Eyelink 1000 Desktop System, SR Research) operating at 1000 Hz. The experiment was implement in Matlab using the Psychophysics Toolbox (Brainard, 1997; Pelli, 1997; Kleiner et al., 2007) and Eyelink Toolbox (Cornelissen et al., 2002) extensions.

#### Stimuli and procedure

**Figure 1.**
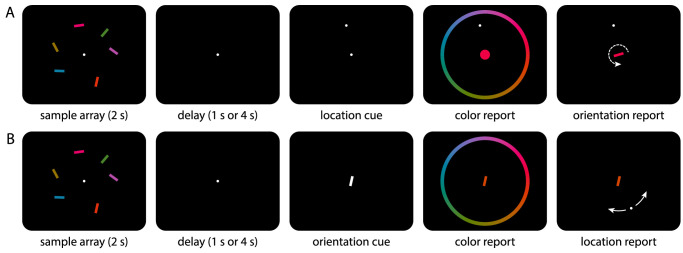
Behavioral tasks with stimulus display and response screens for Experiment 1 (A) and Experiment 2 (B). Stimulus sizes and distances are drawn to scale (except for screen boundaries). The white arrows in the rightmost panels show the possible adjustments of the probe and were not part of the display.

The behavioral task is illustrated in Figure 1A. Each trial began with the presentation of a white fixation point with a radius of 0.25 degree of visual angle (dva), shown at the center of the screen on a black background. The fixation point remained visible throughout the trial until the start of the response phase, and participants were instructed to fixate it while it was present. When stable fixation within a radius of 3 dva was detected, the memory sample array was presented for 2 s.

The sample array consisted of six colored, oriented bars (2 dva × 0.5 dva), located on an invisible circle around the screen center with a radius of 6 dva. Colors were drawn from a color wheel in CIE L*a*b* color space, centered at position (20, 20) in the *ab*-plane, with a radius of 60 and a fixed luminance L = 50. Colors, orientations, and locations of the sample bars were drawn randomly for each trial, with the constraint that color and location angles were separated by at least 20${}^{\circ }$, and orientations differed by at least 10${}^{\circ }$ (given that the space of unique bar orientations covers only 180${}^{\circ }$, a 10${}^{\circ }$ separation was chosen to match the 20${}^{\circ }$ used for color and location).

The presentation of the sample array was followed by a retention interval with a duration of either 1 s (*short delay condition*) or 4 s (*long delay condition*). After this delay, a location cue was shown in the form of a white dot (radius 0.25 dva) at the location where one of the sample items had appeared. Participants had to report first the color, then the orientation of this cued item (in the following referred to as the target item).

A color wheel with a radius of 9 dva appeared once participants started moving the mouse (but no earlier than 0.5 s after cue onset), randomly rotated for each trial. As participants moved the mouse pointer over the color wheel, the fixation point was replaced by a central color probe, a disk with a diameter of 2 dva whose color matched the current selection on the color wheel. Participants made their response by clicking on the color wheel. The color wheel then disappeared and the central probe stimulus changed into a bar while retaining the selected color. Participants rotated this bar by moving the mouse to match the memorized orientation of the target item, and confirmed their response with another mouse click.

Participants completed 6 blocks of 36 trials each (216 trial in total), with delay conditions randomly interleaved and balanced within each block. If participants’ gaze deviated more than 3 dva from the fixation point during the sample or delay period, the present trial was aborted and a new trial with the same delay condition was added to the current block.

### Analysis

#### Model-free analysis

In each trial, we determined the recall errors as the angular deviations of each response from the respective true target feature in circular space (in radians). All orientation values were scaled up by a factor of two to cover the same range [-π, π) as color and location angles. We determined the circular standard deviation (SD) as defined by Fisher (1995) for each participant and each report feature as a model-free measure of recall performance, and used the Pearson correlation coefficient between absolute response errors for the two responses in each trial as a measure of error correlation.

We also determined the deviation of the response from the features of all nontarget items in the same trial to qualitatively evaluate the occurrence of swap errors. Histograms of these nontarget deviations were corrected for the effects of minimum feature separation, by subtracting the expected response distribution around nontargets in the absence of swap errors. The expected response distribution was obtained using a shuffling method, as described in (Schneegans et al. 2021; code available from https://bayslab.com/toolbox). We used the same method to determine the mean absolute deviation (MAD) of response values from the feature values of nontarget items that would be expected in the absence of swap errors. To test for the presence of swap errors, we compared this expected MAD with the actual MAD for each response and task condition.

We adopted the method of Schneegans and Bays (2017) to determine the effect of cue-feature similarity on swap errors. We divided the range of possible absolute distances between items’ feature values within a trial (taking into account minimum feature distance) into eight equal size bins, and assigned each nontarget item to a bin based on its feature distance to the target item in the cue feature dimension (here, location). We then determined the MAD of the response value from the report feature values of the nontarget items separately for each bin.

For statistical comparisons, we used both standard frequentist statistics and Bayesian statistics. For the latter, we report the Bayes factor in favor of the alternative hypothesis, ${\mathrm{BF}}_{10}$.

#### Mixture model fits

To investigate the binding mechanism in the behavioral results, we employed both the joint mixture model fits used by Bays et al. (2011b) and fits with the neural binding model of Schneegans and Bays (2017) in different configurations.

The two-component joint mixture model assumes that responses for each feature are a mixture of target responses, following a von Mises distribution with concentration parameter κ centered on the true target feature value, and uniform responses. Proportions of trials are estimated for the four possible combinations of the mixture components ($\alpha_{\mathrm{TT}}$, $\alpha_{\mathrm{TU}}$, $\alpha_{\mathrm{UT}}$, and $\alpha_{\mathrm{UU}}$, where subscripts T and U indicate target or uniform responses, respectively, for the first and second response). This process yields five free parameters (concentrations $\kappa_{\mathrm{col}}$ and $\kappa_{\mathrm{loc}}$ for color and location, and four mixture proportions that must sum to 1, making one of them redundant). Parameters were estimated separately for each participant by maximum likelihood fits.

Following Bays et al. (2011b), we determine expected mixture proportions for the case of completely independent recall errors as

(1) $$\begin{array}{c} \left[\begin{array}{cc} \alpha_{\mathrm{TT}} & \alpha_{\mathrm{TU}}\\ \alpha_{\mathrm{UT}} & \alpha_{\mathrm{UU}} \end{array}\right]=\left[\begin{array}{c} \alpha_{T•}\\ \alpha_{U}• \end{array}\right]\cdot \left[\begin{array}{cc} \alpha_{•T} & \alpha_{•U} \end{array}\right],\; \end{array}$$

and for the case of perfectly correlated responses as

(2) $$\alpha_{\mathrm{TT}}=\left(\alpha_{T}•+\alpha_{•T}\right)/2\;$$

(3) $$\alpha_{\mathrm{UU}}=\left(\alpha_{U•}+\alpha_{•U}\right)/2$$

(4) $$\alpha_{\mathrm{TU}}=\alpha_{\mathrm{UT}}=0,\;\;$$

where variables of the form $\alpha_{T•}$ indicate the proportion of target or uniform responses in one feature report irrespective of the other one.

The three-component joint mixture model (Bays et al., 2009, 2011b) additionally takes into account nontarget responses or swap errors, in which participants report a feature that was present in the trial's sample array, but does not belong to the cued target item (indicated by subscript N in the mixture proportions). Nontarget responses are assumed to be distributed around the true nontarget features following a von Mises distribution with the same concentration as in target responses. The three mixture components for each response yield nine possible combinations of response types. If a nontarget response occurs for both color and location report, we can further distinguish between matched swap errors, where the *same* nontarget item is selected in both responses (with proportion $\alpha_{\mathrm{NNs}}$), and mismatched swap errors where features of two *different* nontargets are reported ($\alpha_{\mathrm{NNd}}$). This process yields a total of 11 free parameters (two concentration values and ten mixture proportions, one of them redundant).

We also used the two- and three-component mixture models for single features (Luck & Vogel, 1997; Bays et al., 2009) to estimate for each trial the probability each of the two responses was drawn either from the target, nontarget, or uniform component, as described in Schneegans and Bays (2016) (code available from https://bayslab.com/toolbox).

#### Neural binding model fits

**Figure 2.**
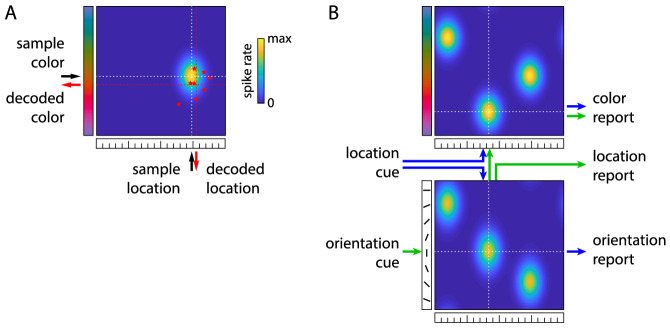
Neural binding model. (A) Encoding and decoding of a single color-location conjunction in neural activity. The conjunctive population code is shown as color-coded activity distribution over the combined space of color hue values and angular locations. Each point in this distribution reflects the combination of preferred feature values for one idealized neuron, and the color reflects the spike rate of that neuron induced by the features of the sample stimulus (black arrows and white dashed lines). Activity is assumed to be maintained over the delay interval, and recall is modeled as maximum likelihood decoding from neural spiking activity. Individual spikes are generated from the spike rates via independent Poisson processes (illustrated as red stars indicating the preferred feature values associated with each spike for an example decoding interval). Noise in the spiking activity introduces deviations of the decoded feature values (red arrows and red dashed lines) from the actual sample features. (B) Architecture of the model variant with spatial binding. Two separate neural populations encode color-location and orientation-location conjunctions, here shown representing features of three sample stimuli. For the location cue task (blue arrows), the location cue is used to select and retrieve the associated features independently from the two populations. For the orientation cue task (green arrows), the orientation cue is used to first retrieve the associated location from one population code, and that location is then used as an intermediary cue to retrieve the associated color.

The neural binding model (Schneegans & Bays, 2017; Lugtmeijer et al., 2021) assumes that feature conjunctions in the memory sample stimuli are encoded in idealized conjunctive neural population codes, in which the firing rate of each neuron is determined by its preferred feature values and associated tuning curves in two feature dimensions (e.g., color and location; Figure 2A). The mean firing rate of neuron i representing the cue feature value $\psi_{j}$ and report feature value $\theta_{j}$ of item j in the sample array is given by

(5) $${\bar{r}}_{i,j}\left(\psi_{j},\theta_{j}\right)=\frac{\gamma }{NM}\phi_{\circ }\left(\psi_{j};\psi_{i}^{'},\kappa_{\psi }\right)\phi_{\circ }\left(\theta_{j};\theta_{i}^{'},\kappa_{\theta }\right)$$

Here, γ is the mean total firing rate of the neural population, which is normalized over the number of items, N, and the number of neurons, M, that contribute to the representation of each item. Neural tuning curves are modeled as von Mises distributions, $\phi_{\circ }$, parameterized with the neuron's preferred feature values, $\psi_{i}^{'}$ and $\theta_{i}^{'}$, and concentration parameters, $\kappa_{\psi }$ and $\kappa_{\theta }$, for the two feature dimensions. Neural spiking activity in the idealized population code is assumed to be generated by independent Poisson processes based on each neuron's mean firing rate, yielding spike counts

(6) $$r_{i,j}∼\mathrm{Pois}\left({\bar{r}}_{i,j}\right).$$

At recall, the feature values of all sample items are decoded through maximum likelihood estimation based on the spiking activity during a fixed decoding interval. The item whose decoded cue feature value is closest to the presented cue is selected, and its decoded report feature value is produced as a response. The derivation of response probability distributions for the population model as used here is described in detail in Lugtmeijer et al. (2021).

We considered different model architectures for the binding between the three feature dimensions in the present experiment (location, color, and orientation). In the *spatial binding model* (Figure 2B), we assume that there are separate conjunctive population codes for the association between each nonspatial feature and its location, implementing the conceptual idea of independent feature maps over visual space. The two responses in each trial are then generated independently from these two populations, such that the probability of reporting a color $\theta_{\mathrm{col}}$ and orientation $\theta_{\mathrm{ori}}$ for a given location cue $\theta_{\mathrm{loc}}$ is determined as

(7) $$p\left(\theta_{\mathrm{col}},\theta_{\mathrm{ori}}|\theta_{\mathrm{loc}}\right)=p\left(\theta_{\mathrm{col}}|\theta_{\mathrm{loc}}\right)p\left(\theta_{\mathrm{ori}}|\theta_{\mathrm{loc}}\right).$$

Two other model variants assume that there is also an explicit population code representation of color-orientation conjunctions. Using this in the present experiment leads to indirect retrieval of one reported features via the other one, but allows direct retrieval of reported features from the cue in Experiment 2. The *binding via color model* assumes that the orientation cue is used to retrieve the item's color based on the location cue, and that color is then used as an intermediary cue to retrieve the associated orientation:

(8) $$p\left(\theta_{\mathrm{col}},\theta_{\mathrm{ori}}|\theta_{\mathrm{loc}}\right)=p\left(\theta_{\mathrm{col}}|\theta_{\mathrm{loc}}\right)p\left(\theta_{\mathrm{ori}}|\theta_{\mathrm{col}}\right)$$

The *binding-via-orientation model* assumes that the location cue is used to first retrieve the item's orientation (although it is reported second), and the color is then retrieved based on the associated orientation:

(9) $$p\left(\theta_{\mathrm{col}},\theta_{\mathrm{ori}}|\theta_{\mathrm{loc}}\right)=p\left(\theta_{\mathrm{ori}}|\theta_{\mathrm{loc}}\right)p\left(\theta_{\mathrm{col}}|\theta_{\mathrm{ori}}\right)$$

All model variants have four free parameters, namely, the overall spike rate γ and a tuning curve width for each feature dimension. The spike rate and tuning curve widths for matching features are shared across the different conjunctive population codes in each model variant. We obtained separate maximum likelihood fits of the three model variants for each participant and delay condition, and compared the quality of fit based on their log-likelihood values. To compare model fits with behavioral data, we simulated responses from the best fitting model, using the same trials as in the actual experiment repeated 100 times, and then applied the same analyses that were used for the behavioral data.

We also compared the best-fitting neural binding model to the two-component joint mixture model using both Akaike information criterion (AIC) and Bayesian information criterion (BIC) scores (because the models differ in the number of free parameters). The three-component joint mixture models performed worse in these scores for all conditions, and we do not report individual comparisons.

### Results

#### Model-free performance measures

**Figure 3.**
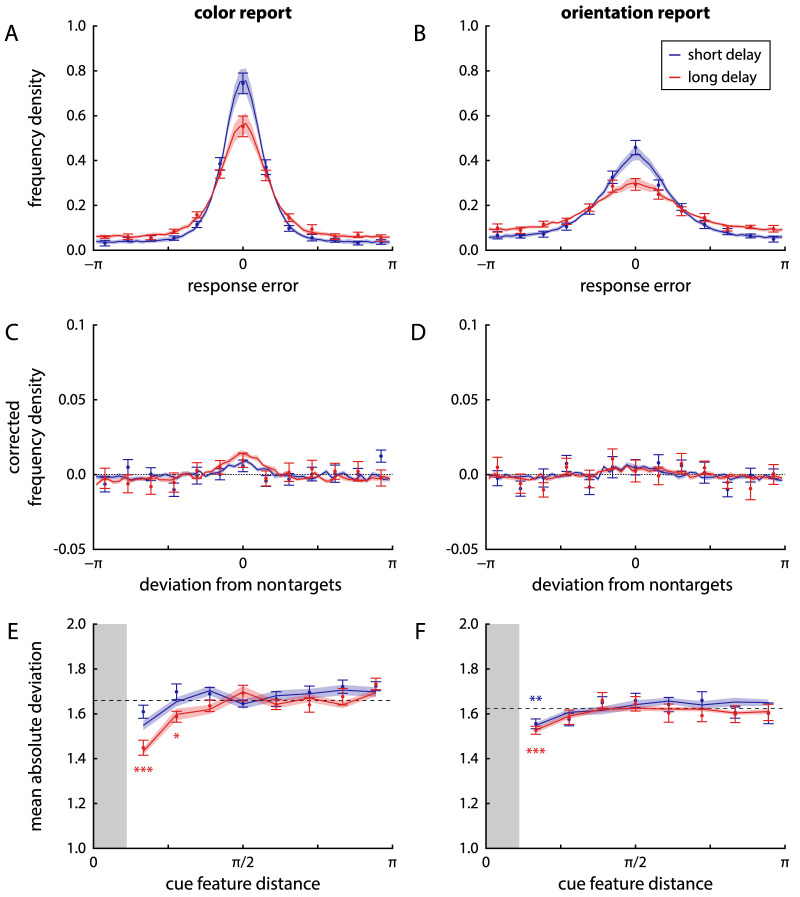
Distributions of response errors and effects of cue similarity in Experiment 1. Points with error bars show behavioral data (mean ± 1 standard error), solid lines and shaded areas show fits from the neural binding model with spatial binding. (A) and (B) Response errors (deviation of reported feature from true target feature) for color and orientation, respectively, shown separately for the short delay condition (blue) and long delay condition (red). (C) and (D) Distribution of response deviations from the feature values of nontarget items in the same trial. The distributions are corrected by subtracting the distributions that would be expected in the absence of swap errors. (E) and (F) MAD of response values from the feature values of nontarget items in the same trial, binned by difference in cue feature between nontarget and target item (with minimum feature distance shown by the shaded gray area). The dashed line indicates the deviation expected by chance, lower values indicate the presence of swap errors. Stars indicate bins in which the MAD is significantly lower than expected by chance (with thresholds 0.05, 0.01 and 0.001 for one, two or three stars, respectively).

Participants viewed an array of six colored, oriented bars, and after a brief delay had to report first the color, then the orientation of one sample item when cued with its location. The duration of the retention interval was varied within blocks of trials, lasting 1 s in the short delay condition and 4 s in the long delay condition. Distributions of response errors for the two reported features and two delay conditions are shown in Figures 3A and B. For both color and location report, recall variability as measured by circular SD increased significantly with longer delay duration (color: from 0.89±0.31 [mean ± SD] for short delays to 1.13±0.24 for long delays, $t_{11}=3.5$, p=0.005, ${\mathrm{BF}}_{10}=10.5$; orientation: from 1.22±0.27 to 1.58±0.29, $t_{11}=10.4$, p<0.001, ${\mathrm{BF}}_{10}=30131$).

There was a small, but statistically significant positive correlation between absolute response errors for the two features reported in each trial, both in the short delay condition (Pearson correlation coefficient r=0.12±0.08, $t_{11}=4.75$, p<0.001, ${\mathrm{BF}}_{10}=62.0$) and the long delay condition (r=0.12±0.12, $t_{11}=3.33$, p=0.007, ${\mathrm{BF}}_{10}=8.2$). The magnitude of this correlation did not differ significantly between delay conditions ($t_{11}=0.22$, p=0.83, ${\mathrm{BF}}_{10}=0.29$).

To detect the occurrence of swap errors, we plotted the distribution of response deviations from the features of nontarget items, with a correction to remove the expected effects of minimum feature distance between the items in each trial (Figures 3C and D). For the color response, the histograms seem to show small central peaks, especially in the long delay condition, suggesting a clustering of responses around nontarget features. A comparison between the MAD of response values from nontarget features and the MAD expected by chance in the absence of swap errors does indeed show a significant difference in the long delay condition (1.63±0.04 vs expected 1.64±0.02, $t_{11}=2.56$, p=0.027, ${\mathrm{BF}}_{10}=2.71$), but not in the short delay condition (1.68±0.04 vs 1.68±0.03, $t_{11}=0.62$, p=0.55, ${\mathrm{BF}}_{10}=0.34$).

For the orientation response, the comparison of observed and expected MAD values likewise showed evidence for a clustering of responses around nontarget feature values in the long delay condition (1.60±0.03 vs 1.61±0.02, $t_{11}=2.74$, p=0.019, ${\mathrm{BF}}_{10}=3.52$), whereas the effect failed to reach significance in the short delay condition (1.62±0.04 vs 1.64±0.02, $t_{11}=2.02$, p=0.069, ${\mathrm{BF}}_{10}=1.31$). It should be noted that in all cases the effect size was very small, and the Bayesian *t* tests showed only relatively weak evidence either in favor or against an effect.

Several previous publications have found that swap errors are more likely to involve nontarget items whose cue feature value is similar to the given cue (Emrich & Ferber, 2012; Souza et al., 2014; Bays, 2016; Oberauer & Lin, 2017). We tested this by grouping items according to their spatial (angular) distance to the target item, and determining the MAD of responses from the report feature values (color and orientation) of the items in each group (Figures 3E and F; Schneegans & Bays, 2017). The occurrence of swap errors should be reflected by a decrease of this MAD below the value expected by chance. In the color response with short delays, we found no evidence for this at any distance (all p>0.067, all ${\mathrm{BF}}_{10}<1.32$), but we found such evidence in the first two distance bins for long delays (first bin: $t_{11}=5.90$, p<0.001, ${\mathrm{BF}}_{10}=282$; second bin: $t_{11}=2.71$, p=0.020, ${\mathrm{BF}}_{10}=3.34$). We also found evidence for swap errors in the first distance bin for the orientation response, both for short delays ($t_{11}=3.19$, p=0.009, ${\mathrm{BF}}_{10}=6.65$) and long delays ($t_{11}=5.35$, p<0.001, ${\mathrm{BF}}_{10}=138$). Taken together, these results indicate that some swap errors did occur for both reported features in this task, but their contribution to overall response errors was quite limited.

#### Mixture model fits

**Figure 4.**
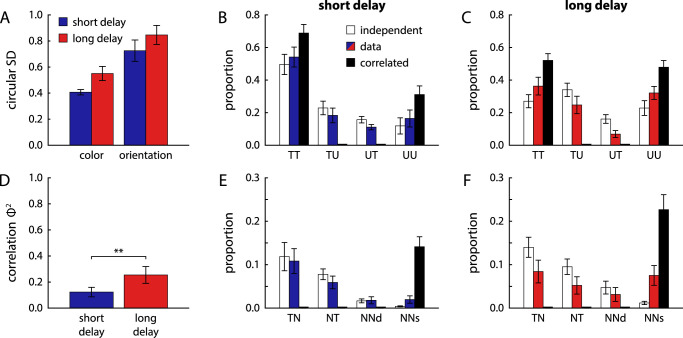
Parameters obtained from joint mixture model fits of response errors in Experiment 1. (A) Circular SD of target components (von Mises distributions) in the two-component joint mixture model fit for the two reported features and two delay conditions. (B) and (C) Estimated proportions of mixture components in the two-component joint mixture model fits, applied separately to the two delay conditions. Components are specified by two letters, T for target and U for uniform, for the response type in the first (color) and second report (orientation). (D) Strength of correlation $\Phi^{2}$ obtained from two-component joint mixture model fits. (E) and (F) Estimated proportions of mixture components in the three-component joint mixture model fits. Only the components including nontarget responses (swap errors) for either of the two features are shown shown, indicated by the letter N. NNd is the mixture component in which two different nontargets items are selected in the two responses, and NNs the mixture component with selection of the same nontarget item.

We used mixture model fits to further elucidate the pattern of response correlations. We fit color and orientation responses in each delay condition with a joint mixture model, which assumes that each response is drawn either from a von Mises distribution centered on the target feature or a uniform distribution. The model fits yield estimates of the precision of target responses for each feature, and of the proportions of possible response combinations (target-target, target-uniform, etc.). The circular SD of target distributions (Figure 4A) was numerically higher in the long delay condition compared with the short delay condition for both features, but the difference was not significant (color: $t_{11}=2.11$, p=0.058, ${\mathrm{BF}}_{10}=1.48$; orientation: $t_{11}=1.42$, p=0.18, ${\mathrm{BF}}_{10}=0.64$).

The proportions of mixture components at short delays (Figure 4B) show that mixed responses, with one report attributed to recall of the target feature but the other attributed to the uniform component, were relatively frequent. In fact, the proportions closely matched those expected for completely independent color and orientation retrieval (white bars), and were inconsistent with predictions from an object-based account in which retrieval of the two features is fully correlated (black bars).

In the long delay condition, estimated proportions of target responses for each feature were reduced, although the difference was only significant for the orientation report ($t_{11}=3.10$, p=0.010, ${\mathrm{BF}}_{10}=5.85$; color: $t_{11}=1.99$, p=0.072, ${\mathrm{BF}}_{10}=1.26$). The overall pattern of estimated mixture proportions was similar to the short delay condition, although there was a modest shift toward more correlated responses. This can be quantified by computing $\Phi^{2}$, a measure of correlation for binary variables, which varies between 0 (uncorrelated) and 1 (perfectly correlated). Although the strength of correlation was relatively low in both delay conditions (Figure 4D), it did increase significantly from short to long delays ($t_{11}=3.91$, p=0.002, ${\mathrm{BF}}_{10}=18.9$).

We additionally fit the data with a three-component joint mixture model that also takes into account swap errors. The overall results of this model fit were consistent with those from the simpler model, and we will focus here on the estimates of nontarget report proportions. An object-based account predicts that if a swap error occurs, the color and orientation of the same nontarget object should be reported. The model fits instead show a predominance of swap errors for only one feature, or swap errors in which nontarget features of two different objects are reported (Figures 4E and F). The mixture proportions in the short delay condition closely matched the pattern for independent memory retrieval for color and orientation, but there was again a modest shift toward more correlated responses in the long delay condition, with a higher tendency to report both features of the same nontarget item. Overall, estimated swap frequencies were low across report features and delays, consistent with the results of the model-free analysis.

**Figure 5.**
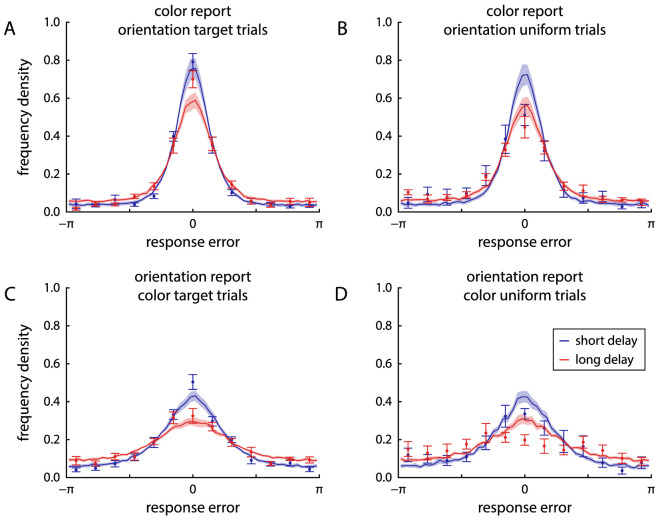
Distribution of response errors for each feature, conditional on the response classification in the other feature. Points with error bars show results for behavioral data, and solid lines and shaded areas show results when the same analysis is applied to simulated data from the neural binding model with spatial binding. (A) Distribution of color response errors in trials in which the orientation response was classified as a target response using a two-component mixture model fit. (B) Distribution of color response errors in trials in which the orientation response was classified as coming from the uniform component of the mixture model. (C, D) Corresponding error distributions for the orientation response in trials in which the color response is classified as coming from the target or uniform mixture component, respectively. For both features and delay conditions, the central peak is reduced, but still present when the response in the other feature is classified as uniform. The reduction is not captured by the model fits.

Finally, we used fits with a two-component mixture model to classify each response as target or uniform (Schneegans & Bays, 2016). Here, we used separate fits for the two reported features to ensure that the classification for one feature would not bias the classification for the other. Figure 5 shows distributions of response errors separately for those trials in which the response in the other feature is classified as target response, and when it is classified as coming from a uniform distribution. For both features and delay conditions, the distribution shows a clear peak even when the response for the other feature was classified as uniform (circular SD lower than expected for uniform distribution, all p<0.001, all ${\mathrm{BF}}_{10}>43.6$; only including participants for which the classification yielded both target and uniform trials, at least 7 out of 12). We did find, however, that the error distributions were somewhat broader when the other response was classified as coming from the uniform component, with significant differences in circular SD for color in the long delay condition ($t_{6}=5.83$, p=0.001, ${\mathrm{BF}}_{10}=37.4$) and orientation in the short delay condition ($t_{11}=3.27$, p=0.007, ${\mathrm{BF}}_{10}=7.52$).

#### Neural binding model

We fit the neural binding model (Schneegans & Bays, 2017) in different configurations to the behavioral data to compare different possible mechanisms of binding between the three visual features used in the task. The spatial binding model that was favored in the previous study assumes that color and orientation are each bound directly and independently to stimulus location, through conjunctive population codes that implement separate feature maps over visual space. For the present task, in which location was used as cue feature, this model predicts that the two reported features should be retrieved independently from the two features maps. As alternatives, we considered two models in which the color and orientation of each sample item are bound directly to each other via a conjunctive population code: In the binding via color model, the location cue is first used to retrieve the target item's color (the feature reported first), and the reported color is then used as an intermediary cue to retrieve the orientation, whereas in the binding via orientation model, the location cue is used to retrieve the orientation, and the reported orientation is used to retrieve the color.

**Figure 6.**
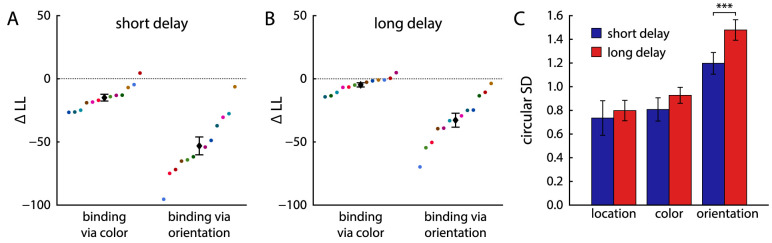
Model comparison between different variants of the neural binding model and estimated decoding precision for each feature. (A) and (B) Difference in log-likelihood (ΔLL) between the model variant with spatial binding and two alternative variants. Negative values indicate better fit for spatial binding, positive values better fit for the alternative. Colored points show differences for individual participants, with colors for each participant fixed across conditions. Black diamonds with error bars shows mean ± 1 standard error. (C) Circular SD of the distribution of decoding errors obtained for each feature in the neural binding model with spatial binding.

We obtained maximum likelihood fits of each of these models, separately for each participant and delay condition. We compared their quality of fit based on the log likelihood, because all models have the same number of free parameters. The results are shown in Figures 6 A and B. The spatial binding model performed better than the two alternatives, providing the best fit for 11 of 12 participants in the short delay condition and 10 of 12 in the long delay condition, with substantially higher log-likelihood values than the binding-via-color model (short delay: ΔLL=14.9±9.3; long delay: ΔLL=4.8±5.8) and the binding-via-orientation model (short delay: ΔLL=53.1±24.5; long delay: ΔLL=32.8±19.3). A single participant's data were fit better by the model variant with binding via color in both delay conditions. However, this participant showed overall poor orientation report performance (reflected by high circular SDs in both delays) and negative correlations in absolute response errors in the long delay condition, the latter being inconsistent with an object-based binding strategy.

The spatial binding model also provided better overall fits than the two-component joint mixture model (short delay: ΔBIC=5.03±6.57, ΔAIC=2.35±6.57, better fit for 10/12 and 8/12 participants, respectively; long delay: ΔBIC=11.1±6.59, ΔAIC=8.42±6.59, better fit for 12/12 and 7/12), indicating that the proposed binding mechanism captures the behavioral data better than the purely descriptive mixture model.

Fits of the spatial binding model to the behavioral data are shown in Figures 3 and 5. As can be seen, the model accurately captures the distributions of response errors and nontarget deviations, as well as the effects of cue similarity on swap errors (Figure 3). The model also qualitatively captures the conditional response distributions in Figure 5 when the same mixture model fit and response classification used for the behavioral data is applied to simulated data from the model. However, because the spatial binding model assumes fully independent retrieval of the two features, it cannot reproduce the slight change in response distributions of one feature depending on the classification of the other response.

The effects of delay condition are captured in the model by an overall decrease in the precision with which features can be decoded from the neural spiking activity (Figure 6C; the decoding precision reflects the tuning curve width for each feature in combination with the global spike rate). The difference in the circular SD of feature decoding was significant for orientation ($t_{11}=9.74$, p<0.001, ${\mathrm{BF}}_{10}=17261$), but not for location ($t_{11}=0.48$, p=0.64, ${\mathrm{BF}}_{10}=0.32$) or color ($t_{11}=2.14$, p=0.056, ${\mathrm{BF}}_{10}=1.53$).

### Discussion

The results in the short delay condition of the present experiment closely replicate the findings of Bays et al. (2011b), demonstrating that these findings are robust to small changes in stimulus display and experimental procedure. In particular, we closely reproduced the estimated proportions of mixture components in the extended mixture model for dual-report data, and found a very similar strength of error correlations

Results of the model comparison with different binding mechanisms implemented in neural populations were consistent with this, favoring a model with direct and independent binding of the two reported features to the location cue over alternative mechanism incorporating direct binding of color to orientation. The spatial binding model captures the finding that swap errors were overall relatively rare—consistent with direct retrieval via the location feature, for which memory is very precise—and that they occurred largely independently for the two reported features. The retrieval of color and orientation from separate feature maps in the model furthermore accounts for nearly independent recall precision in the absence of swap errors, as reflected by the estimated proportions of target and uniform responses in the joint mixture model fits. There was, however, a small degree of positive correlation in the response errors for the two reported features that is not explained by the neural population model.

In the long delay condition, we observed qualitatively similar results in the mixture model fits as in the short delay condition, with an expected decrease in recall precision and proportion of target responses. However, we did observe a significant increase in error correlations between the two responses, albeit on a relatively low level. The neural model comparison also still favored the model variant with independent binding of color and orientation to space, but the advantage over the alternative models was somewhat diminished.

The increase in correlations may indicate that there is at least a partial change in memory format toward a representation more consistent with object files, containing all features of an object bound together. However, a moderate level of response correlations and an increase with longer delays might also be brought about by factors that are not related to the mechanism of feature binding. For instance, variations in global levels of attention from trial to trial would be expected to result in some correlations of response errors, and may have a greater impact when items must be maintained in memory over a longer delay period. We return to the question of how to interpret these results after the second experiment.

## Experiment 2

In Experiment 2, participants had to report the color and location of a sample stimulus when cued with its orientation. This process closely follows one of the task conditions in Experiment 2 of Schneegans and Bays (2017), except that we used larger stimuli and a different mode of response (mouse rather than response dial). We chose a fixed order of responses with color always reported first, because we consider this to be the stronger test of the spatial binding hypothesis. We combined this with the same two delay conditions as in Experiment 1.

### Methods

Twelve new participants (seven female, five male, aged 20–28 years) completed the experiment after giving informed consent. One additional participant was tested, but excluded owing to poor performance (distribution of location response errors not significantly different from uniform as determined by V-test; Fisher, 1995).

The apparatus, stimuli, and procedure were identical to Experiment 1, except that the roles of orientation and location in the task were swapped (Figure 1B). After the presentation of the sample array and the delay period, the central fixation point was replaced by an orientation cue in the form of a white bar with the same dimensions as the sample stimuli, matching the orientation of one bar from the sample array (the target item). Participants reported the color of the target item by clicking on a color wheel as before, with the cue stimulus also acting as color probe. The color wheel was then replaced with a white dot (radius 0.25 dva) that could be moved on the same invisible circle on which the sample stimuli were located. Participants moved this dot using the mouse to report the location of the target item, and confirmed their response with another mouse click.

We applied the same analysis to the data as in Experiment 1. The only difference was that we used a three-component mixture model to classify individual location responses as target, swap or uniform responses. For each trial, we determined the probabilities that the response was based on the target location or each individual nontarget location, or was drawn from a uniform distribution, and classified the trial according to which of these probabilities was highest. For swap trials, this also produced an estimate of which nontarget location was selected in the response.

In the neural binding model, the response probabilities for the spatial binding variant in this task are determined as

(10) $$p\left(\theta_{\mathrm{col}},\theta_{\mathrm{loc}}|\theta_{\mathrm{ori}}\right)=p\left(\theta_{\mathrm{col}}|\theta_{\mathrm{loc}}\right)p\left(\theta_{\mathrm{loc}}|\theta_{\mathrm{ori}}\right),$$

with the location retrieved based on the orientation (and reported in the second response) acting as intermediary cue to retrieve the color. The binding-via-color variant uses the color report as an intermediary cue,

(11) $$p\left(\theta_{\mathrm{col}},\theta_{\mathrm{loc}}|\theta_{\mathrm{ori}}\right)=p\left(\theta_{\mathrm{col}}|\theta_{\mathrm{ori}}\right)p\left(\theta_{\mathrm{loc}}|\theta_{\mathrm{col}}\right),$$

wheras the binding-via-orientation variant allows direct and independent retrieval of both reported features based on the cue,

(12) $$p\left(\theta_{\mathrm{col}},\theta_{\mathrm{loc}}|\theta_{\mathrm{ori}}\right)=p\left(\theta_{\mathrm{col}}|\theta_{\mathrm{ori}}\right)p\left(\theta_{\mathrm{loc}}|\theta_{\mathrm{ori}}\right).$$

### Results

#### Model-free performance measures

**Figure 7.**
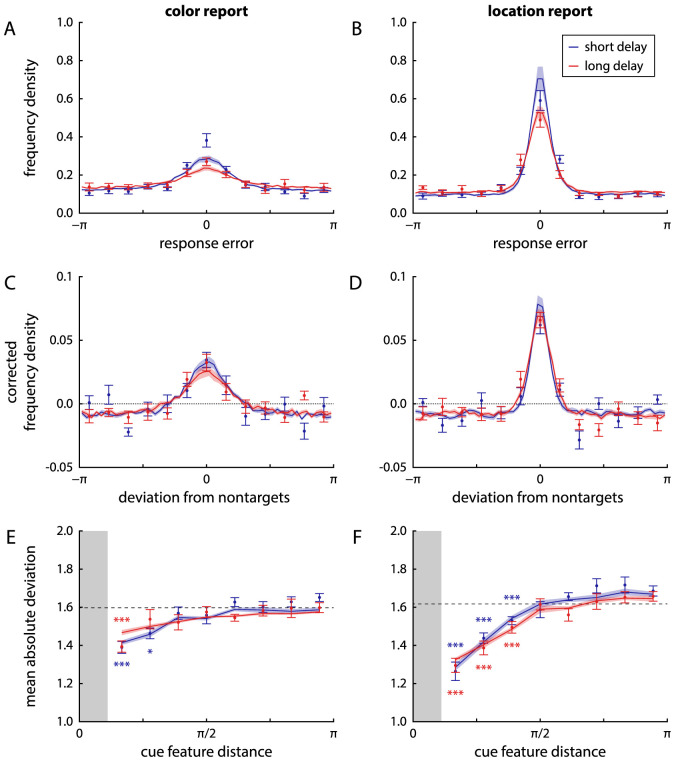
Distributions of response errors and effects of cue similarity in Experiment 2, with fits of the neural binding model with spatial binding, shown in the same format as in Figure 3.

The task in Experiment 2 was identical to that in Experiment 1, except that participants now had to report first the color, then the location of a sample item cued by its orientation. Figures 7A and B show the distributions of response errors for the two reported features and two delay conditions. Circular SD increased significantly from short to long delay duration for both reported features (color: from 1.65±0.22 to 2.01±0.29, $t_{11}=3.36$, p=0.006, ${\mathrm{BF}}_{10}=8.61$; location: from 1.39±0.15 to 1.57±0.21, $t_{11}=3.53$, p=0.005, ${\mathrm{BF}}_{10}=11.0$). Absolute response errors for the two features reported in each trial were significantly correlated in both delay conditions (short delay: r=0.32±0.12, $t_{11}=8.6$, p<0.001, ${\mathrm{BF}}_{10}=5827$; long delay: r=0.24±0.15, $t_{11}=5.05$, p<0.001, ${\mathrm{BF}}_{10}=93$). Although there was a numerical decrease in the level of correlation from short to long delays, the difference was not significant ($t_{11}=2.02$, p=0.069, ${\mathrm{BF}}_{10}=1.30$).

In both reported features, there was a clear clustering of responses around the features of nontarget items, indicating the presence of swap errors (Figures 7C and D). The MAD of response values from nontarget feature values was significantly lower than would be expected by chance for both features and delay conditions (all p≤0.002, all ${\mathrm{BF}}_{10}>24.2$).

There was evidence that swap errors occurred specifically for nontarget items that had a similar orientation as the target, in that the MAD of responses from these nontarget features was lower than expected by chance (Figures 7E and F; color, short delay: p<0.001, ${\mathrm{BF}}_{10}>186$ for first two bins; color, long delay: p<0.001, ${\mathrm{BF}}_{10}=800$ for first bin; location, both delays: p<0.001, ${\mathrm{BF}}_{10}>18$ for first three bins).

#### Mixture model fits

**Figure 8.**
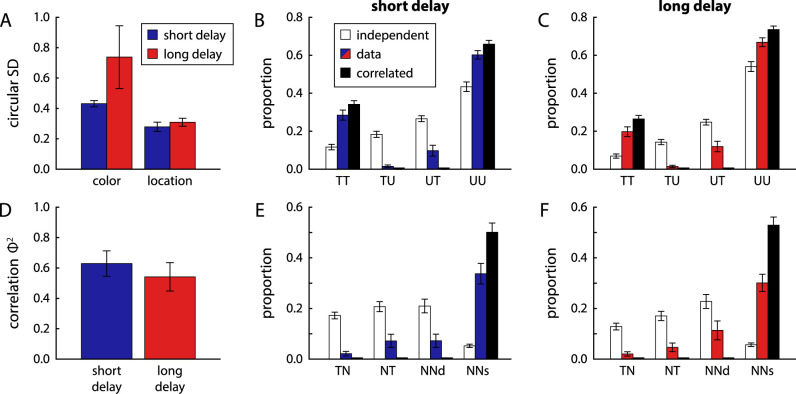
Parameters obtained from joint mixture model fits of response errors in Experiment 2, shown in the same format as in Figure 4. Note that the scale of the *y*-axis in panels (E) and (F) is changed compared with Figure 4.

Figure 8 shows the results of fitting the two responses in each trial with a two-component joint mixture model. There was no significant difference in circular SD of target distributions between delay conditions in either feature (Figure 8A; color: $t_{11}=0.16$, p=0.88, ${\mathrm{BF}}_{10}=0.29$; location: $t_{11}=1.17$, p=0.27, ${\mathrm{BF}}_{10}=0.50$). The proportion of target responses decreased significantly from short to long delay conditions for both the color report ($t_{11}=2.42$, p=0.034, ${\mathrm{BF}}_{10}=2.25$) and the location report ($t_{11}=3.20$, p=0.009, ${\mathrm{BF}}_{10}=6.76$).

The overall pattern of response types obtained from the mixture model fit in this task (Figures 8B and C) differs markedly from that in Experiment 1. In both delay conditions, a large majority of trials show either joint target responses or joint uniform responses for the two reported features, with only a small proportion of trials with mixed reports (predominantly spatial target and color uniform responses). The pattern is close to the prediction for fully correlated retrieval of the two features from memory (black bars). This finding is confirmed by the strength of correlation $\Phi^{2}$ (Figure 8D), which reached substantially higher values than in Experiment 1. The difference in $\Phi^{2}$ between the two delay conditions was not significant ($t_{11}=1.22$, p=0.25, ${\mathrm{BF}}_{10}=0.53$). The fit with a three-component joint mixture model shows a similar pattern (Figures 8E and F). Swap errors occur predominantly in a conjugated form, in which both feature of the same nontarget item are reported. This qualitatively matches the predictions of the fully correlated model.

**Figure 9.**
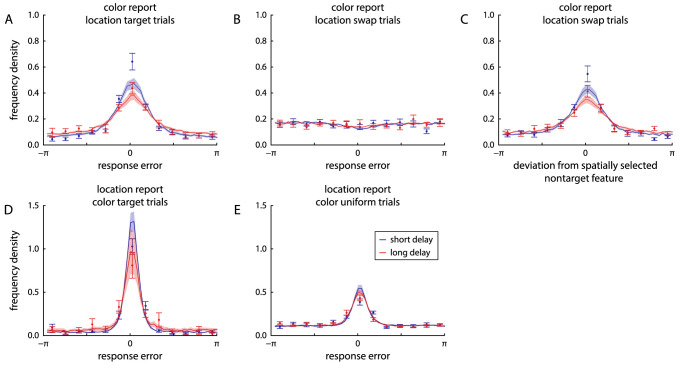
Distribution of response errors for each feature conditional on the response classification in the other feature in Experiment 2. Results are shown in the same format as in Figure 5, but here a three-component mixture model is used to classify location responses. Distributions of color response errors are shown separately for trials with location response classified as coming from the target component (A) or from the nontarget (swap error) component (B). In contrast with the results from Experiment 1, a central peak is no longer detectable in the color response when the location response is classified as swap error. In addition, (C) shows the deviation of the color response from the color of the chosen nontarget item in location swap trials. This panel shows a clearly peaked distribution similar to (A), indicating that participants have a strong tendency to report the color of the spatially selected item independent of whether this selection is correct.

To further illustrate the pattern of response correlations, we used separate mixture model fits to classify the responses in each feature. For the color response, we used a two-component mixture model as before. Because the location response is more precise and location errors tend to be predominantly swap errors (Rajsic & Wilson, 2014; Schneegans & Bays, 2017), we classify these responses using the three-component mixture model, which allows us to estimate which nontarget was selected in swap error trials. The resulting distributions of color response errors show a clear dichotomy between location-target and location-swap trials in both delay conditions (Figures 9A and B). Although the central peak for location-target trials is larger than in the distribution over all trials (Figure 7A), the distribution of color responses in location-swap trials does not show a central peak at all (circular SD not significantly lower than expected for uniform distribution, all p>0.41, ${\mathrm{BF}}_{10}<0.39$). Moreover, if we plot the distribution of color response relative to the color of the nontarget item at the selected location, we see a clear central peak with the same shape as the error distribution in location-target trials (Figure 9C; no significant difference in circular SD for either delay condition, all p>0.09, ${\mathrm{BF}}_{10}<1.06$). This finding demonstrates that, when participants make a swap error in their location response, they will also report the color of the spatially selected item, rather than the color of true target item. This pattern of errors occurs despite the fact that the location response is always made after the color response.

In the distributions of location response errors conditional on the classification of the color report (Figures 9D and E), we likewise find a substantial difference, with a larger central peak for color-target trials and larger tails for color-uniform trials. However, there is still a clear central peak in the color-uniform trials, indicating that a correct location report can still occur in trials with large error in the color response (circular SD significantly lower than expected by chance for both delay conditions, all p<0.001, ${\mathrm{BF}}_{10}>80.0$).

#### Neural binding model

We fit the same variants of the neural binding model to the data as in Experiment 1. However, because the roles of the different stimulus features are now changed, the same models make different predictions. The spatial binding model, in which only color-location and orientation-location conjunctions are explicitly represented, predicts that participants will use the orientation cue to first retrieve the target location, and then use the decoded location as intermediary cue to retrieve the color. The model variant with direct binding of color and location to orientation would predict independent response errors in the current task, whereas the variant with direct binding of the other features to color predicts correlated errors mediated via the first response.

**Figure 10.**
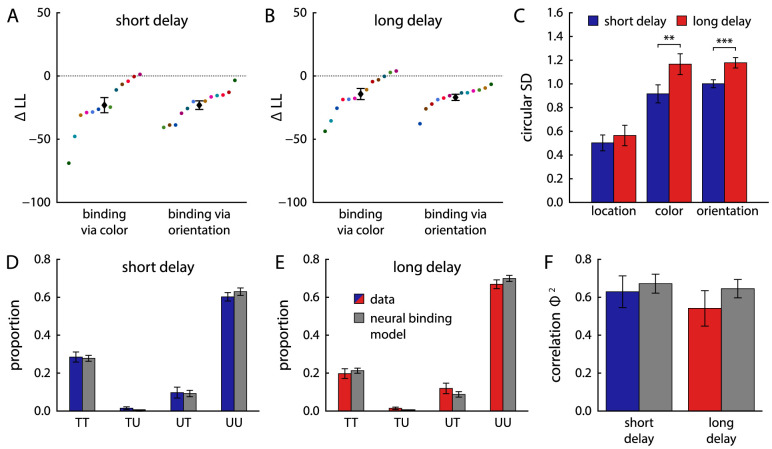
(A–C) Model comparison between different variants of the neural binding model and estimated decoding precision for each feature in Experiment 2, shown in the same format as in Figure 6. (D–F) Estimated mixture proportions and correlation strengths for the two-component joint mixture model applied to simulated data from the neural binding model with spatial binding, compared to estimates for behavioral data.

As expected based on the qualitative results from the mixture model fits, the spatial binding model fit the behavioral data best (Figures 10A and B). It was preferred for 11 (short delay) or 10 (long delay) of 12 participants, respectively, with large difference in log-likelihood relative to the variant with direct binding to the orientation cue (short delay: ΔLL=23.1±20.7; long delay: ΔLL=14.3±15.2) or with binding via color (short delay: ΔLL=23.1±11.8; long delay: ΔLL=16.9±8.51). One participant's data were better fit in both delay conditions by the model variant with direct binding of report features to the orientation cue, but differences in log-likelihood between model fits were small for this participant.

The neural model with spatial binding also captures the data substantially better than the two-component joint mixture model, further supporting this specific binding mechanism (short delay: ΔBIC=103±43.9, ΔAIC=101±43.9; long delay: ΔBIC=76.3±36.5, ΔAIC=73.7±36.5; better fit for all participants in both conditions).

The model provides good fits to the response error distributions (Figures 7A – D), although it deviates slightly from the behavioral data in that it produces less peaked distributions for the color report, and more peaked ones for the location report. This finding can likely be attributed to the simplifying assumption in the model that the location response accurately reflects the decoded location that is used as intermediary cue to retrieve the color. It is plausible that the precision of the reported location is somewhat degraded, owing to the additional delay and possible interference from the color report (which is made by selecting a location on a color wheel). This process would result in the actual location response to be less precise, but the color response to be more precise than predicted in the model (because the color response is based on a more accurate location estimate and therefore contains fewer swap errors).

The neural model with spatial binding accurately captures the conditional error histograms in Figure 9. Because an item's color can only be retrieved via the item's location in this model, a swap error in location will typically be accompanied by a corresponding swap error in the color report (Figures 9A to C). However, errors can still occur in the color retrieval, even when the location of the correct item was selected, such that large color errors are not necessary associated with an incorrect location report (Figures 9D to E). When we apply the fit with a joint two-component mixture to simulated data from the neural binding model, we find that it closely reproduces the estimated mixture proportions as well as the $\Phi^{2}$ values obtained for the behavioral data (Figures 10D to F).

The effects of delay duration are captured in the model by an increased variability in decoding individual features from the neural spiking activity (Figure 10C), with significant increases in circular SD for color ($t_{11}=4.14$, p=0.002, ${\mathrm{BF}}_{10}=26.2$) and orientation ($t_{11}=8.37$, p<0.001, ${\mathrm{BF}}_{10}=4616$), but no significant difference for location decoding ($t_{11}=1.22$, p=0.25, ${\mathrm{BF}}_{10}=0.53$).

### Discussion

The results in both delay conditions of Experiment 2 match those of Schneegans and Bays (2017), and in particular replicate the key qualitative finding of that study: When participants make a swap error in the spatial report, they will typically also report the color of the spatially selected item, with no evidence that they are capable of retrieving the correct target color without the target item's location. This occurs in the present study despite the fact that location was always reported after color, so participants had no incentive to use location as an intermediary cue.

The mixture model analysis produced consistent results, with substantially higher correlations in response errors than in Experiment 1. We note that this qualitative finding is also compatible with object-based memory representations, which would likewise predict correlated response errors. However, fits of the neural population model with binding via space also quantitatively account for the specific proportions of mixture model components estimated for the behavioral data, providing support for this specific binding mechanism.

## General discussion

In two experiments with matched stimuli and procedures, we successfully replicated previous findings from different dual-report delayed reproduction tasks (Bays et al., 2011b; Fougnie & Alvarez, 2011; Fougnie et al., 2013; Schneegans & Bays, 2017; Kovacs & Harris, 2019), and obtained consistent results across different methods of analysis employed in previous studies. This confirms that the different patterns of error correlations observed when using either location or a nonspatial feature as a cue to indicate the target item are indeed a result of the cueing condition, and cannot be attributed to other differences in stimulus display or procedure between the previous studies.

We did not observe any qualitative changes in the pattern of error correlations when we substantially increased the duration of the retention interval, indicating that there was no overall change in the format of working memory, as had been suggested in previous studies using change detection tasks (Treisman & Zhang, 2006; Logie et al., 2011). The correlation patterns were well explained by a neural model in which nonspatial features of an object are bound to each other only indirectly via their shared location in visual space (Schneegans et al., 2016; Schneegans & Bays, 2017).

The neural binding model used here is based on the general assumption that conjunctions of features are encoded in the spiking activity of neural populations, and that recall errors arise from noise in the neural activity. The model combines aspects of resource models (with a fixed total spike rate in the neural population limiting capacity) and the slots-plus-averaging model (with individual spikes acting as discrete samples that are averaged when retrieving a feature; Schneegans et al., 2020). In explaining recall errors as an effect of noise in memory representations, it is also related to the recently proposed target confusability competition model (Schurgin et al., 2020), which has been shown to closely correspond with a specific formulation of the neural population model (Bays, 2019). However, the neural binding model considers noise not only in the feature to be reported, but also in the cue feature used to indicate which item is the target, and explains swap errors and imprecision of responses as a result of the same memory variability. In this regard, it is similar in turn to the interference model of Oberauer and Lin (2017).

In the present experiment, the implementation of the neural binding model with purely spatial binding was preferred for a large majority of participants across experiments and delay conditions, and only few individual participants showed patterns of response errors that were better captured by different binding mechanisms. It is plausible that these cases were merely the result of noise in the behavioral data, although we acknowledge that the relatively small sample size used here does not allow us to reliably assess individual differences in feature binding. Another recent study using a dual-report paradigm did observe some flexibility in the binding mechanism (Schneegans et al., in press). In particular, it found that presentation time could take over the role of space in mediating binding between other features when sample items were presented sequentially at the same location, and that a mix of binding strategies may be used when stimuli are separated both in time and space (see also Heuer & Rolfs, 2021). However, this study likewise did not find any evidence for object-based representations as basis for feature binding, supporting the view that binding via space is the default mechanism when memorizing simultaneously displayed sample arrays.

One deviation in the behavioral data from the predictions of the spatial binding model was that recall errors when using a location cue were not entirely independent, but showed a low level of positive correlations, and this correlation was increased in the long delay condition. This observation would be consistent with at least a quantitative change in visual working memory toward a representational format such as slots or object files, in which all features of an item are inherently bound to each other without a special role of location. However, the orientation cue task in Experiment 2 showed no evidence for a reduced role of location in mediating binding between other features.

We therefore believe that these correlations are more likely to be the result various factors that are not directly linked to the binding mechanism, and which are not taken into account in the neural binding model. One of these is trial-to-trial variations in vigilance, that is, the level of attention to the task. Lapses of attention have been proposed as a cause for large recall errors in conditions with low memory demand (Zhang & Luck, 2008), and recent work has shown that trial-to-trial variations in attention affect working memory performance (deBettencourt et al., 2019). In the present task, lapses in attention would be expected to cause errors in both responses within a trial, leading to the observed correlations. It has furthermore been proposed that fluctuations in attention specifically affect the successful maintenance of memory items over long delay periods (Hakim et al., 2020), which would explain the observed increase in error correlations (together with an overall increase in the estimated proportion of uniform responses) in the long delay condition of Experiment 1.

In addition, different levels of spatial attention to individual items within a trial would also induce error correlations without object-based memory representations. It is well established that participants can prioritize individual items in a trial, either due to stimulus properties (Bays & Husain, 2008; Rajsic et al., 2016) or an explicit incentive to do so (Bays et al., 2011a; Gorgoraptis et al., 2011). The neural binding model as used here assumes that resources are always distributed evenly across items, and variability in recall precision is purely due to stochastic processes (Schneegans et al., 2020), but this is likely an over-simplification.

Correlations owing to differences in attention could be reinforced over the retention interval through rehearsal, in particular if rehearsal makes use of similar space- or object-based attentional processes in working memory as in perception (Kong & Fougnie, 2019; Grieben et al., 2020). This would result in increasing correlations in memory strength between memorized features of different items, even if these features are held in independent stores (such as separate feature maps).

Finally, verbal (re-)coding of stimuli (e.g., blue horizontal bar) may contribute to memory performance and may create a form of bound object representation outside of visual memory stores. Fougnie and Alvarez (2011) used articulatory suppression in their experiments and obtained very similar qualitative results as Bays et al. (2011b) and the present study. However, the additional time available in the long delay condition may increase the use of verbal coding and its impact on recall errors.

A recent study by Sone et al. (2021), using a dual-report task with location cues, sought to determine whether the sequential report of different features affected the outcome, and tested a task variant with simultaneous report of colors and orientations in a combined response display. The study assessed correlations by grouping trials according to absolute response error in one feature, and measuring how closely the responses in the other feature was concentrated around the correct target value. This method yielded clear evidence for error correlations between the two features both for sequential and simultaneous reports, with the latter revealing stronger correlations than the former. The authors interpreted this as evidence for object-based representations in memory.

We applied the same analysis to the present results, and found that correlations in the present Experiment 1 (Supplementary Figure S1) were noticeably lower than those observed by Sone et al. with the same set size and delay, both for simultaneous and sequential report. However, we found substantially higher correlations than this previous study in the present Experiment 2 (Supplementary Figure S2). In particular, for those trials with the highest location errors, color report performance was at chance levels (consistent with our model-based analysis). In contrast, performance was still substantially above chance levels for all trial bins in the results of Sone et al., suggesting that what they observed was an intermediate level of correlation not consistent with the form of object-based, all-or-nothing memory representations proposed by slot models.

A possible basis for the discrepant results between our Experiment 1 and the findings of Sone et al. (2021) lies in the various differences in stimuli and procedure between the two studies, such as the shorter sample presentation time of 0.5 s in the previous study compared to 2 s in the present experiments. We note that recall performance in the closest matching condition of Sone et al. (2021) was overall lower than in the present Experiment 1 (Supplementary Figure S1 vs. Figure 7 from Sone et al.). Greater task difficulty may incentivize participants to focus attention on a subset of items, as has been suggested for a previous study finding high error correlations (Gajewski & Brockmole, 2006; Peich et al., 2013).

Sone et al. (2021) further aimed to distinguish object-based representations from location-based binding of features, using sample arrays with pairs of colored, oriented triangles of different sizes centered at the same location (with the smaller triangles completely within the boundaries of the larger ones). Performance was better when participants had to remember two features of the same triangles (either the smaller or the larger ones) than when they had to memorize, for example, the colors of the larger and the orientations of the smaller triangles, and performance in the latter condition was not significantly different from a condition in which the features of all items had to be memorized. Based on this, the authors concluded that working memory stores whole objects, with no special role for location.

One limitation of this experiment is that it does not distinguish object-based effects in perception and memory. Because the stimuli are still presented quite briefly (0.5 s), it is plausible that the encoding stage is the limiting factor for performance in this task. Moreover, although the stimuli in the task are centered at the same point, participants are still likely to focus their attention on different parts of the display when they try to memorize the different-sized stimuli. It is true, however, that fully overlapping stimuli—which occur, for instance, when they are presented sequentially at the same location—are a challenge for models relying exclusively on binding via space. The recent study of Schneegans et al. (in press) has shown that in such cases, presentation time or temporal order rather than location can mediate binding between other features.

We further note that one of the change detection studies that motivated the present work (Logie et al., 2011) also observed disruptive effects on performance when a nonspatial feature (color or shape) was changed while it was task-irrelevant, but these effects only occurred at shorter delays (0 s or 0.5 s) than they did for location changes. Results from a subsequent study indicate that these effects are driven by a visual transient signal that occurs in the absence of a delay and extends to very short delay periods, but can be eliminated by introducing a visual pattern mask (Bocincova et al., 2017).

We have not attempted in the present study to explicitly model the causes of decreasing performance with longer retention intervals. A previous study extended the original neural population model (Bays, 2014) to implement different mechanisms of memory deterioration over time (Schneegans & Bays, 2018) and found support for random drift in population activity as the likely mechanism. Here, we confined ourselves to assessing the effects of delay duration with separate model fits for the different task conditions. The model fits show that delay effects in the present study can be explained by an overall increase in the variability of decoded feature values. This finding is consistent with the drift hypothesis, but also compatible with other mechanisms (e.g. Barrouillet et al., 2007).

One limitation of the dual-report paradigm is that it can only elucidate binding mechanisms between features that participants are instructed to memorize for later report. In particular, location was always a task-relevant feature, in contrast with the change detection tasks for color-shape binding used in previous studies (Treisman & Zhang, 2006; Logie et al., 2011). Therefore, we cannot rule out that participants in these tasks use specific strategies that decrease the disruptive effects of location changes. These may include approaches alluded to before, such as verbal coding or focussing attention on a subset of items, but also changes in memory representations that are yet to be specified.

However, we believe the current results demonstrate that there is no automatic change in the format of working memory representations with longer retention intervals. The continuity in the observed patterns of error correlations across delay conditions shows that there is merely a gradual decrease in performance, but no qualitative change in the mechanism of feature binding. A neural architecture with separate feature maps over visual space therefore not only captures visual processing during perception and early stages of memory, but offers a viable explanation for the organization of visual working memory in general.

## Supplementary Material

Supplement 1

Supplement 2
