# Supplementary material for: Mechanisms of feature binding in visual working memory are stable over long delays
Source: J Vis. 2021 Nov 16;21(12):7. doi: 10.1167/jov.21.12.7 (PMC8606872; doi:10.1167/jov.21.12.7)
Supplement: Supplement 1 [file jovi-21-12-7_s001.pdf]

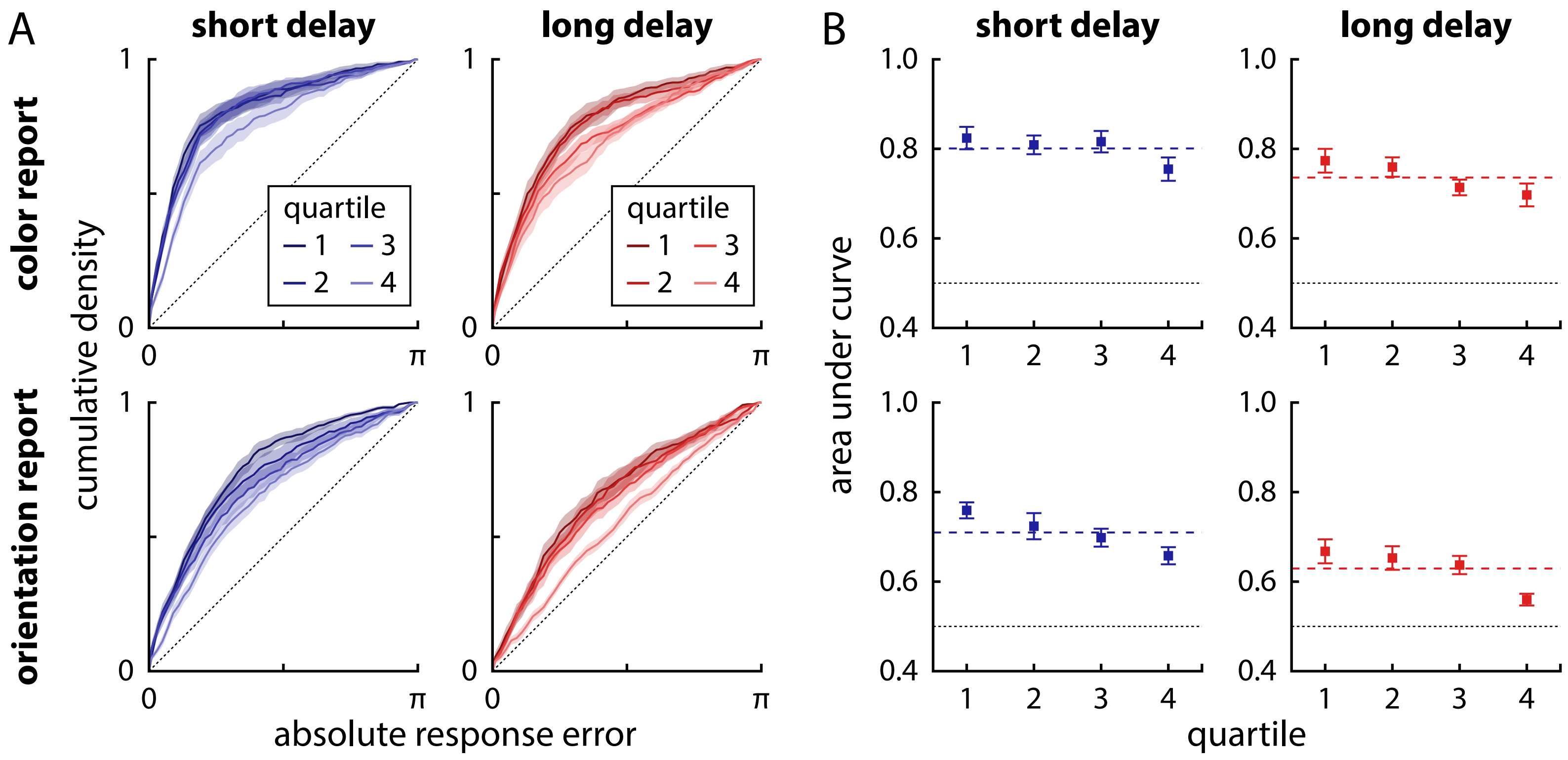

Figure S1: Results of the analysis from Sone et al. (2021) applied to data from Experiment 1. This model-free analysis is based on binning trials according to the absolute response error in one reported feature, and then determining the area under the curve of cumulative absolute response errors for the other reported feature. (A) Cumulative distribution of absolute response errors for each reported feature and delay condition, shown separately for quartiles of trials according to absolute response error in the other feature (ordered from lowest to highest errors). The black dotted line (main diagonal) is the cumulative distribution expected by chance (i.e., if participants were purely guessing). (B) Area under the curve of cumulative distributions, scaled so that possible areas range form 0 to 1. Data points show areas for each quartile (mean ± 1SE), the dashed line is the area under the curve over all trials (averaged across participants), and the black dotted line indicates the value expected by chance. See Sone et al. (2021) for method details.
