# Supplementary material for: Mechanisms of feature binding in visual working memory are stable over long delays
Source: J Vis. 2021 Nov 16;21(12):7. doi: 10.1167/jov.21.12.7 (PMC8606872; doi:10.1167/jov.21.12.7)
Supplement: Supplement 2 [file jovi-21-12-7_s002.pdf]

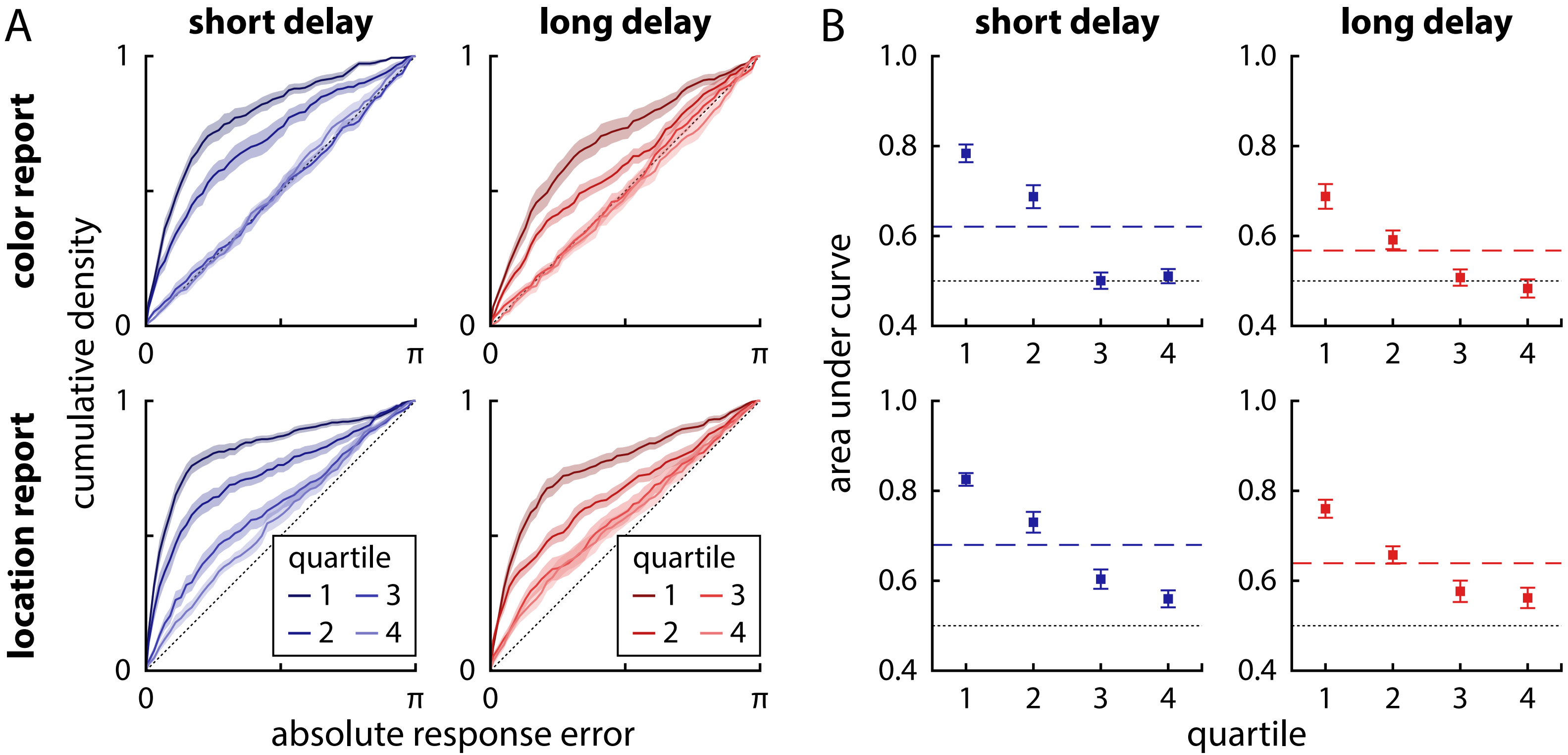

Figure S2: Results of analysis from Sone et al. (2021) applied to data from Experiment 2, shown in the same format as in Figure S1.
